# A standardised nomenclature for long non‐coding RNAs

**DOI:** 10.1002/iub.2663

**Published:** 2022-07-26

**Authors:** Ruth L. Seal, Susan Tweedie, Elspeth A. Bruford

**Affiliations:** ^1^ HUGO Gene Nomenclature Committee, European Molecular Biology Laboratory European Bioinformatics Institute, Wellcome Genome Campus Hinxton UK; ^2^ Department of Haematology University of Cambridge School of Clinical Medicine Cambridge UK

**Keywords:** gene names, gene symbols, human genes, lncRNA, standardisation

## Abstract

The HUGO Gene Nomenclature Committee (HGNC) is the sole group with the authority to approve symbols for human genes, including long non‐coding RNA (lncRNA) genes. Use of approved symbols ensures that publications and biomedical databases are easily searchable and reduces the risks of confusion that can be caused by using the same symbol to refer to different genes or using many different symbols for the same gene. Here, we describe how the HGNC names lncRNA genes and review the nomenclature of the seven lncRNA genes most mentioned in the scientific literature.

**Abbreviations**
ABCA9‐AS1: ABCA9 antisense RNA 1
CIBAR1‐DT: CIBAR1 divergent transcript
COSMOC: cell fate and sterol metabolism associated divergent transcript of MOCOS
CPMER: cytoplasmic mesoderm regulator
DUBR: DPPA2 upstream binding RNA
ECRAR: endogenous cardiac regeneration‐associated regulator
FAM182A: family with sequence similarity 182 member A
FAM182B: family with sequence similarity 182 member B
GAS1RR: GAS1 adjacent regulatory RNA
GREP1: glycine rich extracellular protein 1
GTL2: gene trap locus 2
H19: H19 imprinted maternally expressed transcript
HAO2‐IT1: HAO2 intronic transcript 1
HGNC: HUGO Gene Nomenclature Committee
HOTAIR: HOX transcript antisense RNA
HOXC11: homeobox C11
HOXD: homeobox D
HUGO: Human Genome Organisation
IGF2: insulin like growth factor 2
LINC02998: long intergenic non‐protein coding RNA 2998
lncRNA: long non‐coding RNA
LNX: ligand of numb‐protein X
MALAT1: metastasis associated lung adenocarcinoma transcript 1
mascRNA: MALAT1‐associated small cytoplasmic RNA
MEG3: maternally expressed gene 3
MEG8: maternally expressed gene 8
MEG9: maternally expressed gene 9
MEN1: menin 1
MIR17HG: miR‐17‐92a‐1 cluster host gene
MIR675: microRNA 675
MIR7‐3HG: MIR7‐3 host gene
MTLN: mitoregulin
MYC: MYC proto‐oncogene, bHLH transcription factor
NBDY: negative regulator of P‐body association
NCBI: National Center for Biotechnology Information
NEAT1: nuclear paraspeckle assembly transcript 1
NEAT2: nuclear enriched abundant transcript 2
NIHCOLE: ncRNA involved in NHEJ oncogenic ligation efficiency
NXTAR: negative expression of androgen receptor regulating lncRNA
ORF: open reading frame
PCA3: prostate cancer associated 3
PCBP2‐OT1: PCBP2 overlapping transcript 1
PINCR: p53‐induced noncoding RNA
PTTG1: PTTG1 regulator of sister chromatid separation, securin
PVT1: Pvt1 oncogene
RB1: RB transcriptional corepressor 1
RENO1: regulator of early neurogenesis 1
SNHG3: small nucleolar RNA host gene 3
TINCR: TINCR ubiquitin domain containing
TncRNA: telomeric ncRNA
TncRNA: tiny ncRNA
TncRNA: trophoblast noncoding RNA
TP53: tumor protein p53
TRPS1‐AS1: TRPS1 antisense RNA 1
VINC: virus inducible non‐coding RNA
XIST: X inactive specific transcript

## 1 INTRODUCTION

The HUGO (Human Genome Organisation) Gene Nomenclature Committee (HGNC) is the only group with the official capacity to name human genes. We name protein‐coding genes, pseudogenes and non‐coding RNA (ncRNA) genes; our commentary on our latest nomenclature guidelines^1^ and our recent comprehensive review^2^ discuss how we name different types of ncRNA genes, including lncRNA, microRNA and small nucleolar RNA genes. Each gene with official nomenclature approved by the HGNC is assigned a gene **symbol**, a corresponding descriptive gene **name** and a unique **HGNC ID**. Gene symbols allow unambiguous discussion about genes and are recorded as previous symbols if any updates to the nomenclature are made, while HGNC IDs are associated with the underlying gene sequence and hence are stable unless the gene structure changes substantially (e.g., split into more than one gene or merged with another gene).

The naming of lncRNA genes is currently the main focus of our ncRNA naming work, in part due to the large numbers of these genes annotated in the human genome, and in part due to the many papers being published on the lncRNAs encoded by these genes. LncRNA genes are the only class of human genes, other than protein‐coding (pc) genes, where research groups may suggest a symbol based on a function or important characteristic of the gene. The HGNC encourages research groups to contact us prior to publication to ensure that proposed symbols meet with HGNC guidelines.^1^ Briefly, new human gene symbols should not clash with existing vertebrate gene symbols, commonly used abbreviations, or common English words; symbols should contain only uppercase Latin letters and Arabic numerals; symbols should not contain references to any species; symbols must not be pejorative or offensive. The use of punctuation is avoided although hyphens may be used in specific cases. Unique symbols have always been important to aid literature searching but are now more necessary than ever with the advent of text mining. HGNC curators search the scientific literature for papers on lncRNA genes; where published symbols do not fulfil HGNC guidelines we contact authors to discuss suitable alternatives. For this reason, we approved the unique symbol *CHROMR*, “cholesterol induced regulator of metabolism RNA,” for the lncRNA gene first published as *CHROME* ^3^ and *EMSLR*, “E2F1 mRNA stabilising lncRNA,” for the lncRNA first published as *EMS*.^4^ Both *CHROME* and *EMS* are poor search terms, and “chrome” is a widely used English word.

**FIGURE 1:**
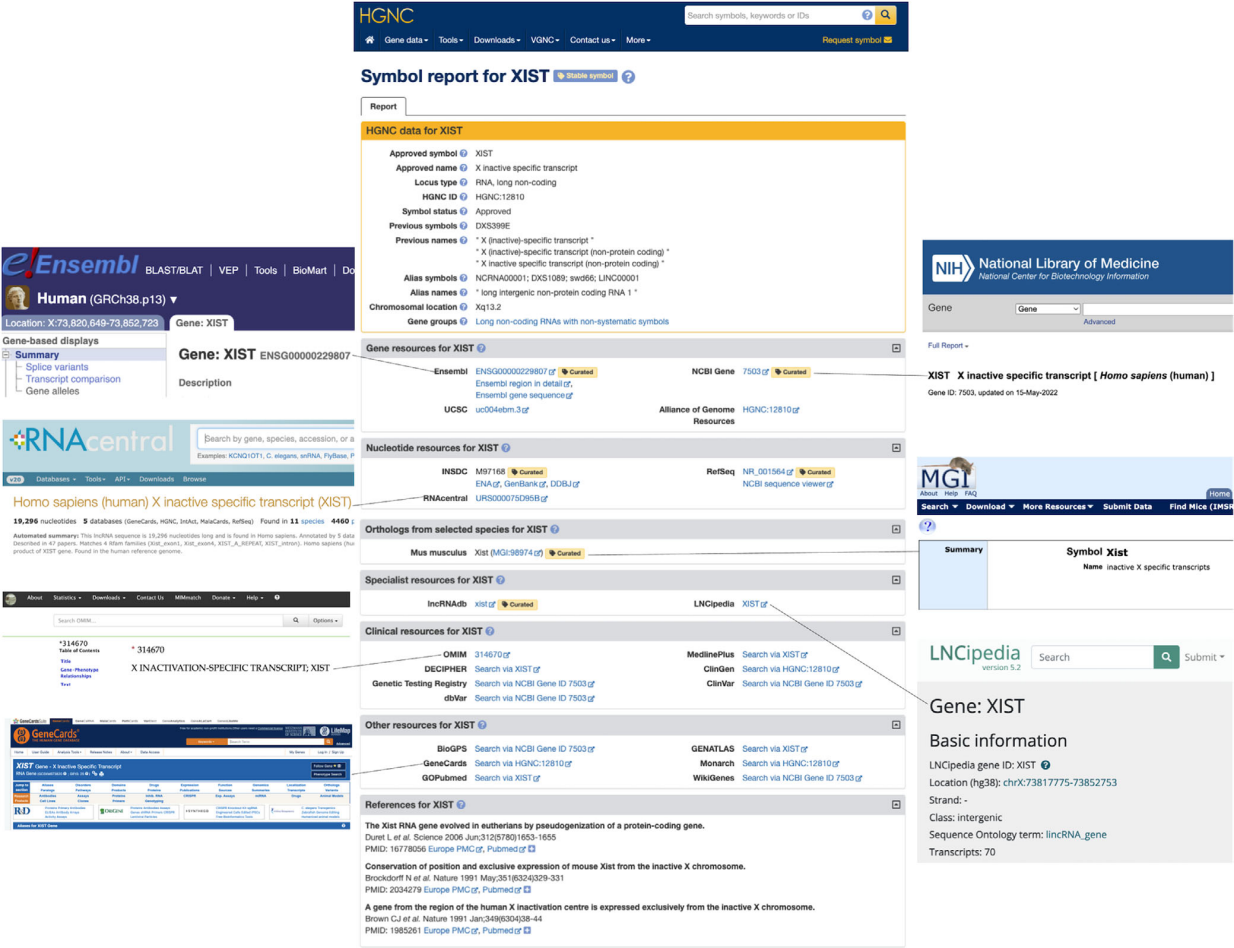
An example Symbol Report for the lncRNA gene *XIST* from genenames.org. HGNC Symbol Reports present the HGNC‐approved gene symbol, gene name, unique HGNC ID and other manually curated data in the top HGNC data section. The “Stable symbol” luggage tag is shown at the top of the report for approved symbols which are unlikely to ever be changed. Further down the report, links to many different biomedical resources are provided. Here, we have highlighted the resources that are particularly relevant to lncRNAs

**FIGURE 2:**
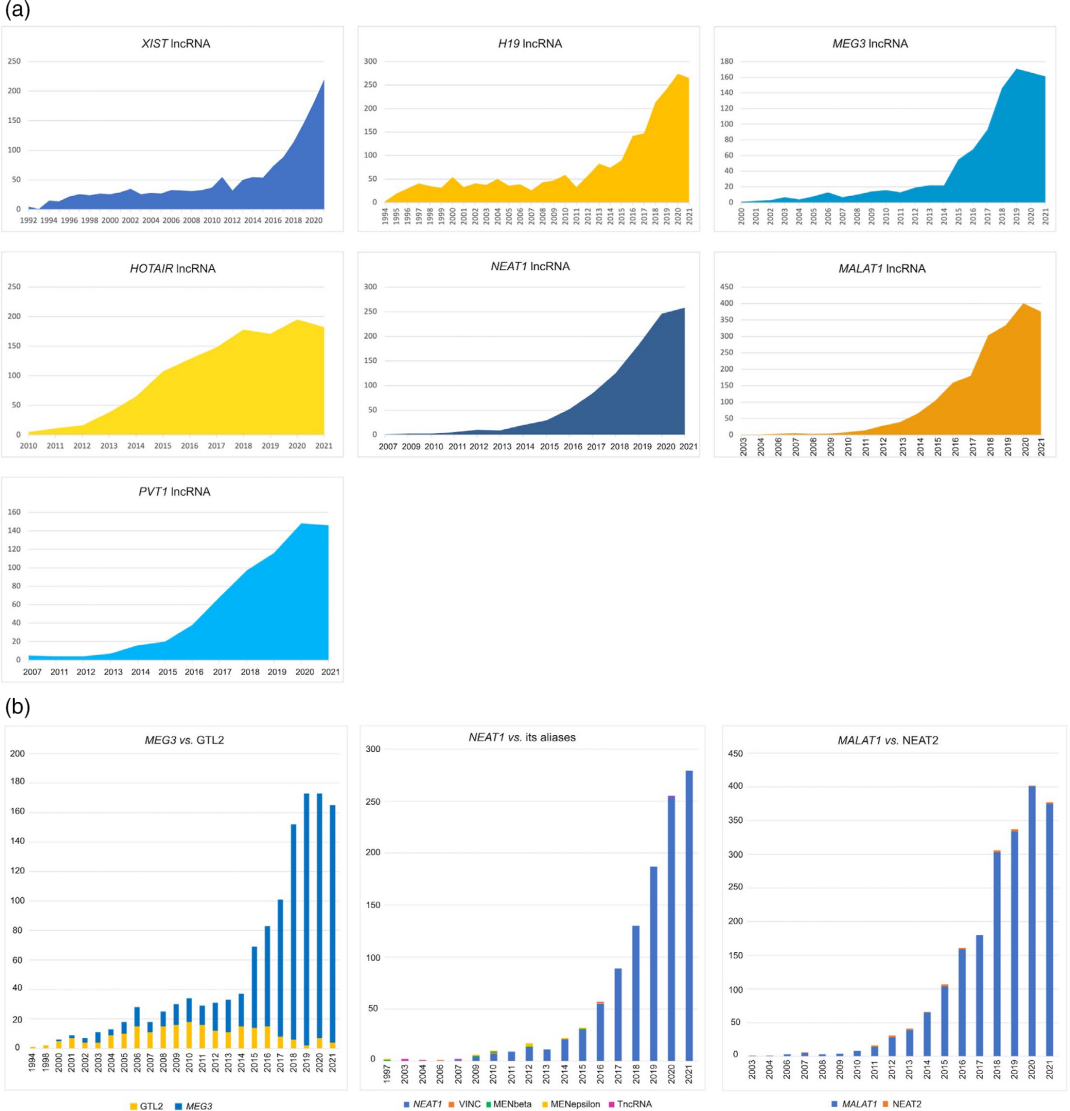
The number of publications in PubMed for the top seven most highly published lncRNA genes. (a) For each of the seven highly published lncRNA genes, the number of publications has rapidly increased over the last 5 years. (b) For all of the most highly published lncRNA genes, the majority of publications use the current HGNC approved symbol. The first chart shows how over time the number of publications supporting the approved symbol *MEG3* have increased compared to the previous symbol GTL2. The second chart shows *NEAT1* and its published aliases (VINC, MENbeta, MENepsilon, TncRNA); the usage of *NEAT1* far surpasses any of its aliases within the last decade. The third chart compares usage of the approved symbol *MALAT1* and its published alias NEAT2; again *MALAT1* is highly supported. The other four most highly published lncRNA symbols have negligible numbers of publications that do not use the approved symbol

The HGNC has been naming lncRNA genes since the early 1990s but it is within the last decade that this endeavour has taken up a large proportion of our gene naming effort. HGNC approved lncRNA gene symbols are displayed in relevant biomedical resources such as Ensembl,^5^ NCBI Gene,^6^ RNAcentral,^7^ LNCipedia,^8^ OMIM^9^ and GeneCards.^10^ The HGNC provides a Symbol Report on our website (genenames.org) for each gene with an approved symbol that features links out to these and other relevant biomedical resources; Figure 1 shows an example Symbol Report for *XIST*. Where there is a mouse ortholog, we provide a link to the relevant page of the Mouse Gene Database.^11^ Figure 2a demonstrates how rapidly the number of publications has increased with time for the seven most widely published lncRNA genes. We have provided a summary of the nomenclature of each of these seven lncRNAs below. These examples illustrate many of the typical issues we consider while naming genes.

## 2 XIST

The *XIST* (X inactive specific transcript) gene was first published in 1991^12^ and the symbol was approved by the HGNC in the same year. As of April 2022, there were over 1,900 hits in PubMed for the XIST symbol (Figure 2a) with no other competing gene symbols in general use and no overlapping use of the abbreviation to refer to different concepts. *XIST* is conserved in eutherians and contains two exons derived from a pseudogene that has a coding ortholog from the *LNX* (ligand of numb‐protein X) family, published as Lnx3, at a conserved position in birds, reptiles and amphibians. However, the majority of *XIST* exons contain sequence derived from mobile elements that is completely unrelated to the pseudogene.^13^, ^14^ *XIST* is necessary for inactivation of one X chromosome in cells with two copies of this chromosome; please see^15^ for a recent review on the mechanisms by which *XIST* achieves this. Notably, the *XIST* sequence element known as “Repeat A” that has been shown to be necessary for gene silencing is not located within the pseudogene‐derived sequence.^14^

## 3 H19

The *H19* symbol was approved by the HGNC in April 1994 based on^16^ who stated that “Despite the fact that it is transcribed by RNA polymerase II and is spliced and polyadenylated, we suggest that the *H19* RNA is not a classical mRNA. Instead, the product of this unusual gene may be an RNA molecule.” The *H19* symbol is also approved for the mouse and rat orthologs; in all three species this lncRNA gene shows sequence similarity and hosts the microRNA gene *MIR675* in an exon. The symbol *H19* should be viewed as historical as it does not represent a characteristic or function of the gene; this is an example of a gene symbol that the HGNC will retain as it is supported by the lncRNA community and widely published (Figure 2a). *H19* originates from a paper on mouse fetal‐specific hepatic mRNAs and the assumption is that the “H” stood for hepatic although this is not explicitly stated; this paper already commented that *H19* is expressed in heart and skeletal muscle.^17^ The original HGNC‐approved gene name that accompanied the *H19* symbol was “H19, imprinted maternally expressed untranslated mRNA” but this has since been updated to “H19 imprinted maternally expressed transcript” because the term mRNA is now used only for genes that produce transcripts which are translated into protein. *H19* is expressed in the foetus and placenta; the current approved name reflects the fact that this imprinted gene is expressed from the maternal allele. This is in contrast with the neighbouring protein coding gene *IGF2*, which is also highly expressed in the placenta but is expressed from the paternal allele.^18^ *H19* is found in some adult tissues such as skeletal muscle and the adrenal gland, and its dysregulation has been associated with many types of cancer although there are contrasting theories about its involvement in the progression of these cancers.^19^

## 4 MEG3

*MEG3* (maternally expressed gene 3) is another maternally imprinted lncRNA gene. This gene was originally approved with the symbol GTL2 (gene trap locus 2) based on the identification of the mouse ortholog from the site of a gene trap integration.^20^ It was subsequently renamed to *MEG3* to be grouped with other maternally imprinted genes using the MEG# root symbol^21^ in mouse and human ‐ *MEG8* and *MEG9* are approved symbols for other lncRNA genes. Like *H19*, *MEG3* has been associated with many types of cancer and has been reported to be a tumour suppressor gene via regulation of *TP53*,^22^ by separate regulation of RB1,^23^ and by suppression of angiogenesis.^24^ Figure 2b shows usage of GTL2 versus *MEG3* over time and shows how *MEG3* is now the symbol supported by the lncRNA community. The GTL2 symbol has been retained in the *MEG3* entry as a “previous symbol” in line with HGNC's normal practise of retaining all previously approved gene symbols.

## 5 HOTAIR

*HOTAIR* (HOX transcript antisense RNA), which lies antisense to the protein coding *HOXC11* gene, was approved in 2007 based on.^25^ This lncRNA was initially reported to regulate genes at the HOXD locus. It has since been reported as positively regulating *HOXC11* levels in *cis* and negatively regulating HOXD in *trans*, perhaps due to a duplicated noncoding element within the *HOTAIR* gene and HOXD locus.^26^ This lncRNA has also been associated with many types of cancer.^27^ *HOTAIR* has a mouse ortholog named *Hotair*, and mouse models have been reported with contrasting phenotypes.^28^ We now have a more systematic way of reporting genes that are antisense to protein coding genes (see the “Systematic protocol” section below), and the symbol “*HOTAIR*” could be considered somewhat frivolous which we avoid where possible, but we will retain the *HOTAIR* symbol due to overwhelming usage.

## 6 NEAT1

Two transcripts produced by the *NEAT1* gene were first published as MENbeta and MENepsilon in a paper about the transcript map surrounding the *MEN1* locus,^29^ but these two transcripts were not further characterised at that time. The *NEAT1* gene is over 620 kb downstream from the *MEN1* gene, with many intervening protein coding genes between these two loci, and it has not been associated with the *MEN1* gene functionally, so a symbol linking this gene to *MEN1* is not optimal. A short transcript from the *NEAT1* locus was described as “trophoblast noncoding RNA” (TncRNA)^30^ but this isoform is not found in the mouse ortholog (*Neat1*) and “TncRNA” is not unique as it is also used as an abbreviation for both “telomeric ncRNA” and “tiny ncRNA” so would not be a suitable gene symbol. Additionally, the longer isoforms of *NEAT1* are widely expressed so nomenclature linking this gene specifically to the trophoblast would be misleading. The symbol *NEAT1* was first used in a study that identified large noncoding RNAs displaying nuclear enrichment.^31^ The name accompanying the symbol was “​​nuclear enriched abundant transcript 1,” which has been recorded as a gene name alias by the HGNC. The HGNC were contacted in 2009 by a researcher writing a review on this gene who requested that *NEAT1* could be approved for the human gene and *Neat1* for the mouse ortholog. The HGNC coordinates with the Mouse Genomic Nomenclature Committee wherever possible to approve equivalent nomenclature for mouse and human orthologs. At that time the human and mouse transcripts had been shown to be necessary for the formation of paraspeckles in the nucleus,^32^ and therefore the HGNC agreed upon a name that reflected this function and that could be approved alongside the *NEAT1* symbol: “nuclear paraspeckle assembly transcript 1.” *NEAT1* also has the alias VINC (virus inducible non‐coding RNA) based on its detection in mouse brains infected with Japanese encephalitis virus or Rabies virus.^33^ As can be seen from Figure 2b, the *NEAT1* symbol is overwhelmingly supported by the research community over any of its aliases.

## 7 MALAT1

*MALAT1* (metastasis associated lung adenocarcinoma transcript 1) was first identified in a study to find differences in gene expression between tumours of non‐small cell lung cancer that metastasised and those that did not.^34^ *MALAT1* is located close to *NEAT1* in the genome of both human and mice and is highly expressed in both species. *MALAT1* is localised to nuclear speckles and hence has been given the alias NEAT2,^31^ but unlike *NEAT1* it is not required for assembly of paraspeckles. The NEAT2 alias is far less published than *MALAT1* (Figure 2b). The *MALAT1* locus also produces a small cytoplasmic tRNA‐like transcript via tRNA processing ribonucleases known as mascRNA (MALAT1‐associated small cytoplasmic RNA).^35^ Although not restricted to lung cancers, overexpression of *MALAT1* has been associated with metastasis in several different types of cancer,^36^ though a smaller number of studies have reported that the lncRNA has a tumour suppressor role in some cancers. As the *MALAT1* symbol is very well supported, the HGNC has no plans to change this symbol, but we would consider updating the accompanying descriptive gene name in the future to something more informative, if there is community support to do so.

## 8 PVT1

The *PVT1* symbol was first used for the mouse ortholog (*Pvt1*) following its discovery as the major locus for murine plasmacytoma variant translocations.^37^ The human ortholog was subsequently found in Burkitt's lymphoma translocations.^38^ The HGNC originally approved the gene name “pvt‐1 (murine) oncogene homolog” as the descriptive name accompanying the approved *PVT1* symbol, but we have since updated this to the simpler name “Pvt1 oncogene,” which reflects how this gene is described in many papers. The HGNC no longer references other species in gene names to reduce possible confusion. Studies have reported that the *PVT1* promoter regulates the *MYC* gene, and that presence of the *PVT1* transcript is not necessary for this function.^39^ The *PVT1* gene hosts several microRNA genes and has widely been reported to be able to compete for binding of microRNAs.^40^ Because it is a microRNA host locus, it also has the alias symbol MIR1204HG based on the most 5′ miRNA gene in the locus. The *PVT1* symbol is highly published and is unique to this gene.

## 9 MORE RECENT EXAMPLES OF lncRNA SYMBOLS APPROVED BASED ON PUBLICATIONS

We hope that many of our more recently‐approved lncRNA gene symbols will achieve the same level of support as the above symbols in the scientific literature in the future. Recent examples of approved lncRNA gene symbols that reflect the function of the encoded lncRNA include *RENO1* for “​​regulator of early neurogenesis 1,”^41^ *COSMOC* for “cell fate and sterol metabolism associated divergent transcript of MOCOS”^42^ and *CPMER* for “cytoplasmic mesoderm regulator.”^43^ All of these symbols were agreed with the HGNC prior to publication. We were able to approve the symbol *NXTAR* post publication^44^ but we updated the gene name, with the agreement of the authors, from the published name “next to androgen receptor” to the more functionally informative name “negative expression of androgen receptor regulating lncRNA,” which still fits with the *NXTAR* symbol.

## 10 THE HGNC “STABLE” TAG

As outlined in the HGNC guidelines,^1^ we are now committed to keeping the symbols of clinically relevant genes as stable as possible, and minimising changes to well‐published gene symbols. In the era of clinical genomics, it is impossible to contact all clinicians, patient groups, charities and interested individuals to inform them of symbol changes, so it is important that the symbols of genes referred to in the clinic are kept as stable as possible. HGNC curators are currently working through a list of clinically relevant genes and adding a “stable” tag onto the Symbol Reports for these genes once curators are satisfied that the approved symbols are appropriate and are unlikely to be changed (see the top of the *XIST* Symbol Report shown in Figure 1). We have added this tag to over 40 non‐coding RNA genes to date, including the two clinically relevant lncRNA genes, *MIR17HG* and *PCA3*. *MIR17HG* has been associated with Feingold syndrome type 2 as shown in the GenCC (Gene Curation Coalition,^45^) database, while there is now a clinical test that evaluates levels of *PCA3* RNA to help assess prostate cancer risk.^46^ We have also added the stable tag to the seven highly published lncRNA genes described above, as we have no plans to change these symbols.

## 11 SYSTEMATIC PROTOCOL FOR NAMING ANNOTATED HUMAN lncRNA GENES

**FIGURE 3:**
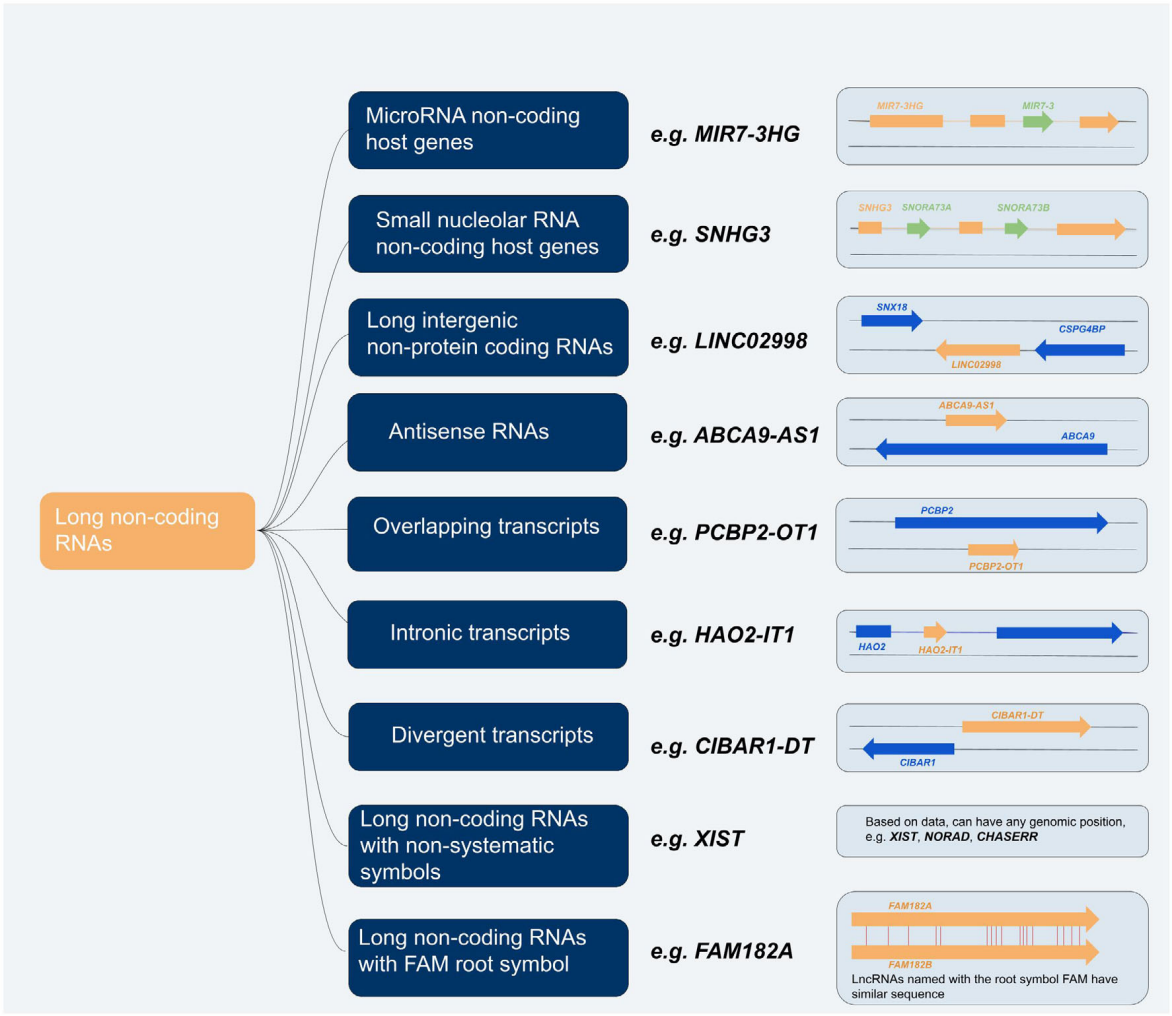
The eight categories used by the HGNC to name lncRNA genes. For each category an example is given, along with a diagram that demonstrates, where applicable, how lncRNA genes within these categories are named relative to other genes. LncRNA genes are shown in pale orange, small RNAs are shown in green, and protein coding genes are shown in blue. Each category can be browsed from the HGNC gene group page “Long non‐coding RNAs” (https://www.genenames.org/data/genegroup/#!/group/788)

In addition to approving lncRNA symbols based on published data, the HGNC has a systematic protocol for naming lncRNA genes that have been manually annotated by the RefSeq annotators at the National Center for Biotechnology Information (NCBI)^6^ and/or the GENCODE annotators at Ensembl.^5^ Note that the HGNC has a large set of unnamed lncRNA genes to work through; we currently prioritise genes that are mentioned in publications but have no suitable information for a non‐systematic symbol, and lncRNA genes that have been annotated by both of the above‐mentioned manual annotation projects. The eight categories, along with the non‐systematic category based on published data described above, used for this systematic naming are shown in Figure 3. Please also see the decision‐making chart published as fig. 1 in Reference 1 and a more detailed description of each lncRNA naming category in Reference 2.

The eight systematic categories of lncRNA genes are as follows:

- if an lncRNA gene hosts a microRNA gene in an exon or intron it is named as a microRNA non‐coding host gene with the symbol format [microRNA symbol]HG, for example, *MIR7‐3HG*
- if an lncRNA gene hosts a small nucleolar (sno)RNA gene it is named as a small nucleolar RNA non‐coding host gene with the root symbol SNHG for example, *SNHG3*
- if an lncRNA gene is intergenic with respect to protein‐coding genes it is named as a long intergenic non‐protein coding RNA with the root symbol LINC followed by a unique five digit number, for example, *LINC02998*
- if an lncRNA gene overlaps the genomic span of a pc gene but is located on the opposite strand compared to that pc gene it is named as an antisense RNA with the symbol format [pc symbol]‐AS suffixed with a unique number, for example, *ABCA9‐AS1*
- if an lncRNA gene overlaps at least one exon of a pc gene on the same strand, it is named as an overlapping transcript with the symbol format [pc symbol]‐OT suffixed with a unique number, for example, *PCBP2‐OT1*
- if an lncRNA is contained within an intron of a pc gene it is named as an intronic transcript with the symbol format [pc symbol]‐IT suffixed with a unique number, for example, *HAO2‐IT1*
- if an lncRNA gene shares a bidirectional promoter with a pc gene it is named as a divergent transcript with the symbol format [pc symbol]‐DT for example, *CIBAR1‐DT*
- if an lncRNA gene has another lncRNA paralog in the human genome, these paralogs may be named with the FAM root symbol (family with sequence similarity), for example, *FAM182A* and *FAM182B*. Note that the FAM root symbol is also used for pc genes, but these can be distinguished via locus type.

Although the above protocol is applied where no other suitable information is available at the time of naming, these symbols can become well‐established in the literature and so may not necessarily be updated when further data are published, unless there is agreement between research groups working on the genes to do so. Where there is an ortholog in other species, the HGNC may pursue a rename in order that the orthologs be approved with the same symbol and name. For example, the human lncRNA gene *DUBR* (DPPA2 upstream binding RNA) had the previous symbol *LINC00883*, while mouse gene *Dubr* had the previous symbol *5330426P16Rik*.

## 12 A CAUTIONARY NOTE ON THE IMPORTANCE OF APPROVED GENE NOMENCLATURE

During our literature searches for papers on lncRNA genes, HGNC curators have noticed that many papers continue to use names based on BAC clones in the human genome assembly, which were used in previous versions of the Ensembl website as symbols, or primary identifiers, for lncRNAs. These clone‐based identifiers used to be displayed on Ensembl gene reports for human genes that had no HGNC symbol, but have now been removed completely and are not searchable in the current version of the Ensembl website. We recently found the following examples in the literature: AF178030.2^47^ which has the approved HGNC symbol *TRPS1‐AS1*; RP11‐138 J23.1,^48^ which has the approved symbol *NIHCOLE*; RP11‐276H19.1^49^ has approved symbol *GAS1RR*; and RP3‐326I13.1^50^ which has the approved symbol *PINCR*. We urge researchers to check genenames.org ahead of publication to see if there is an approved symbol for an lncRNA gene. Although HGNC symbols are used as the primary gene labels in Ensembl, the most recently approved symbols may be missing due to differences in database update cycles, so it is always worth checking genenames.org using the Ensembl gene ID (ENSG#) if Ensembl does not display an approved gene symbol. Where no HGNC symbol is available, researchers should contact the HGNC prior to publication to request a new symbol using our online gene symbol request form (https://www.genenames.org/contact/request/) that is linked from the header of every page of our website.

## 13 PROTEIN CODING GENES THAT WERE PREVIOUSLY ANNOTATED AS lncRNA GENES

It may be surprising to consider that most lncRNA genes contain open reading frames (ORFs) but these are usually short in length, unsupported by conservation in other species, lack structural features such as protein domains, and are not supported by peptides from mass spectrometry. Post annotational experimental evidence may show that such ORFs are translated and therefore the locus types of lncRNA genes may be updated to protein coding. The following genes were updated based on published data: *MTLN* ‐ mitoregulin^51^ has the previous symbol *LINC00116*; *GREP1* ‐ glycine rich extracellular protein 1^52^ was previously *LINC00514*; *NBDY* ‐ negative regulator of P‐body association^53^ was previously *LINC01420*. Although the HGNC will usually rename such genes, particularly if a new symbol is proposed by authors, in some cases we may retain the gene symbol and only update the gene name. This is the case for the gene *TINCR* as this is a well‐published symbol that has been retained, while the locus is now annotated as protein coding. The gene name is now “TINCR ubiquitin domain containing” in place of the previous gene name “tissue differentiation‐inducing non‐protein coding RNA.” The *TINCR* symbol is also still used in papers discussing the protein.^54^, ^55^ Note that there are still many recent papers describing *TINCR* as an lncRNA; it is possible that this gene has both coding and non‐coding isoforms but this is true for many protein‐coding genes and merits discussion. The HGNC does not approve separate symbols for non‐coding isoforms of protein‐coding genes, for example, ECRAR (endogenous cardiac regeneration‐associated regulator)^56^ is listed as an alias of the protein coding *PTTG1* gene because ECRAR represents a non‐coding variant.

## 14 GROUPING TRANSCRIPTS TOGETHER AS lncRNA GENES

For protein‐coding genes the presence of ORFs provides information to gene annotators on when a set of overlapping transcripts should be grouped into the same gene or split into different genes. There is no equivalent information for lncRNA genes, which means that criteria need to be agreed upon between different annotation groups as to when transcripts should be grouped together as an lncRNA gene and when they should not. The HGNC plans to host a workshop on this subject with annotation groups and selected lncRNA researchers to decide upon guidelines for this issue. We hope that this will result in consistent grouping of transcripts into lncRNA gene models in the future.

## 15 CONCLUSION

The field of lncRNA research continues to grow rapidly each year. Consistent use of approved gene symbols for lncRNA genes will mean that all research papers and associated online resources are easily searchable for lncRNAs. We encourage researchers publishing on new lncRNA genes to contact the HGNC prior to submission. This will enable HGNC curators to check that the proposed symbol follows our guidelines and will prevent changes to gene symbols post publication. HGNC‐approved symbols appear on our website, www.genenames.org, and in many key lncRNA resources.

## CONFLICT OF INTEREST

The authors have no conflicts of interest to report.

## References

[1] Bruford EA , Braschi B , Denny P , Jones TEM , Seal RL , Tweedie S . Guidelines for human gene nomenclature. Nat Genet. 2020;52:754–758. 32747822 10.1038/s41588-020-0669-3 PMC7494048

[2] Seal RL , Chen L‐L , Griffiths‐Jones S , et al. A guide to naming human non‐coding RNA genes. EMBO J. 2020;39:e103777. 32090359 10.15252/embj.2019103777 PMC7073466

[3] Hennessy EJ , van Solingen C , Scacalossi KR , et al. The long noncoding RNA CHROME regulates cholesterol homeostasis in primate. Nat Metab. 2019;1:98–110. 31410392 10.1038/s42255-018-0004-9 PMC6691505

[4] Wang C , Yang Y , Zhang G , et al. Long noncoding RNA EMS connects c‐Myc to cell cycle control and tumorigenesis. Proc Natl Acad Sci U S A. 2019;116:14620–14629. 31262817 10.1073/pnas.1903432116 PMC6642410

[5] Frankish A , Diekhans M , Jungreis I , et al. GENCODE 2021. Nucleic Acids Res. 2021;49:D916–D923. 33270111 10.1093/nar/gkaa1087 PMC7778937

[6] O'Leary NA , Wright MW , Brister JR , Ciufo S , Haddad D , et al. Reference sequence (RefSeq) database at NCBI: Current status, taxonomic expansion, and functional annotation. Nucleic Acids Res. 2016;44:D733–D745. 26553804 10.1093/nar/gkv1189 PMC4702849

[7] RNAcentral Consortium . RNAcentral 2021: Secondary structure integration, improved sequence search and new member databases. Nucleic Acids Res. 2021;49:D212–D220. 33106848 10.1093/nar/gkaa921 PMC7779037

[8] Volders P‐J , Anckaert J , Verheggen K , et al. LNCipedia 5: Towards a reference set of human long non‐coding RNAs. Nucleic Acids Res. 2019;47:D135–D139. 30371849 10.1093/nar/gky1031 PMC6323963

[9] Amberger JS , Bocchini CA , Scott AF , Hamosh A . OMIM.org: Leveraging knowledge across phenotype‐gene relationships. Nucleic Acids Res. 2019;47:D1038–D1043. 30445645 10.1093/nar/gky1151 PMC6323937

[10] Stelzer G , Rosen N , Plaschkes I , et al. The GeneCards suite: From gene data mining to disease genome sequence analyses. Curr Protoc Bioinformatics. 2016;54:1.30.1–1.30.33. 10.1002/cpbi.5 27322403

[11] Bult CJ , Blake JA , Smith CL , et al. Mouse genome database (MGD) 2019. Nucleic Acids Res. 2019;47:D801–D806. 30407599 10.1093/nar/gky1056 PMC6323923

[12] Brown CJ , Ballabio A , Rupert JL , et al. A gene from the region of the human X inactivation Centre is expressed exclusively from the inactive X chromosome. Nature. 1991;349:38–44. 1985261 10.1038/349038a0

[13] Elisaphenko EA , Kolesnikov NN , Shevchenko AI , et al. A dual origin of the Xist gene from a protein‐coding gene and a set of transposable elements. PLoS One. 2008;3:e2521. 18575625 10.1371/journal.pone.0002521 PMC2430539

[14] Duret L , Chureau C , Samain S , Weissenbach J , Avner P . The Xist RNA gene evolved in eutherians by pseudogenization of a protein‐coding gene. Science. 2006;312:1653–1655. 16778056 10.1126/science.1126316

[15] Loda A , Collombet S , Heard E . Gene regulation in time and space during X‐chromosome inactivation. Nat Rev Mol Cell Biol. 2022;23:231–249. 35013589 10.1038/s41580-021-00438-7

[16] Brannan CI , Dees EC , Ingram RS , Tilghman SM . The product of the H19 gene may function as an RNA. Mol Cell Biol. 1990;10:28–36. 1688465 10.1128/mcb.10.1.28-36.1990 PMC360709

[17] Pachnis V , Belayew A , Tilghman SM . Locus unlinked to alpha‐fetoprotein under the control of the murine raf and Rif genes. Proc Natl Acad Sci U S A. 1984;81:5523–5527. 6206499 10.1073/pnas.81.17.5523 PMC391738

[18] Rachmilewitz J , Goshen R , Ariel I , Schneider T , de Groot N , Hochberg A . Parental imprinting of the human H19 gene. FEBS Lett. 1992;309:25–28. 1380925 10.1016/0014-5793(92)80731-u

[19] Alipoor B , Parvar SN , Sabati Z , Ghaedi H , Ghasemi H . An updated review of the H19 lncRNA in human cancer: Molecular mechanism and diagnostic and therapeutic importance. Mol Biol Rep. 2020;47:6357–6374. 32743775 10.1007/s11033-020-05695-x

[20] Schuster‐Gossler K , Bilinski P , Sado T , Ferguson‐Smith A , Gossler A . The mouse Gtl2 gene is differentially expressed during embryonic development, encodes multiple alternatively spliced transcripts, and may act as an RNA. Dev Dyn. 1998;212:214–228. 9626496 10.1002/(SICI)1097-0177(199806)212:2<214::AID-AJA6>3.0.CO;2-K

[21] Miyoshi N , Wagatsuma H , Wakana S , et al. Identification of an imprinted gene, Meg3/Gtl2 and its human homologue MEG3, first mapped on mouse distal chromosome 12 and human chromosome 14q. Genes Cells. 2000;5:211–220. 10759892 10.1046/j.1365-2443.2000.00320.x

[22] Zhu J , Liu S , Ye F , et al. Long noncoding RNA MEG3 interacts with p53 protein and regulates partial p53 target genes in Hepatoma cells. PLoS One. 2015;10:e0139790. 26444285 10.1371/journal.pone.0139790 PMC4596861

[23] Kruer TL , Dougherty SM , Reynolds L , et al. Expression of the lncRNA maternally expressed gene 3 (MEG3) contributes to the control of lung cancer cell proliferation by the Rb pathway. PLoS One. 2016;11:e0166363. 27832204 10.1371/journal.pone.0166363 PMC5104461

[24] Liu J , Li Q , Zhang K‐S , et al. Downregulation of the Long non‐coding RNA Meg3 promotes angiogenesis after ischemic brain injury by activating notch signaling. Mol Neurobiol. 2017;54:8179–8190. 27900677 10.1007/s12035-016-0270-z PMC5684256

[25] Rinn JL , Kertesz M , Wang JK , et al. Functional demarcation of active and silent chromatin domains in human HOX loci by noncoding RNAs. Cell. 2007;129:1311–1323. 17604720 10.1016/j.cell.2007.05.022 PMC2084369

[26] Nepal C , Taranta A , Hadzhiev Y , et al. Ancestrally duplicated conserved noncoding element suggests dual regulatory roles of HOTAIR in cis and trans. iScience. 2020;23:101008. 32268280 10.1016/j.isci.2020.101008 PMC7139118

[27] Zhang J , Liu X , You L‐H , Zhou R‐Z . Significant association between long non‐coding RNA HOTAIR polymorphisms and cancer susceptibility: A meta‐analysis. Onco Targets Ther. 2016;9:3335–3343. 27330313 10.2147/OTT.S107190 PMC4898434

[28] Selleri L , Bartolomei MS , Bickmore WA , et al. A Hox‐embedded long noncoding RNA: Is it all hot air? PLoS Genet. 2016;12:e1006485. 27977680 10.1371/journal.pgen.1006485 PMC5157941

[29] Guru SC , Agarwal SK , Manickam P , et al. A transcript map for the 2.8‐Mb region containing the multiple endocrine neoplasia type 1 locus. Genome Res. 1997;7:725–735. 9253601 10.1101/gr.7.7.725 PMC310681

[30] Geirsson A , Lynch RJ , Paliwal I , Bothwell ALM , Hammond GL . Human trophoblast noncoding RNA suppresses CIITA promoter III activity in murine B‐lymphocytes. Biochem Biophys Res Commun. 2003;301:718–724. 12565840 10.1016/s0006-291x(03)00028-7

[31] Hutchinson JN , Ensminger AW , Clemson CM , Lynch CR , Lawrence JB , Chess A . A screen for nuclear transcripts identifies two linked noncoding RNAs associated with SC35 splicing domains. BMC Genomics. 2007;8:39. 17270048 10.1186/1471-2164-8-39 PMC1800850

[32] Clemson CM , Hutchinson JN , Sara SA , et al. An architectural role for a nuclear noncoding RNA: NEAT1 RNA is essential for the structure of paraspeckles. Mol Cell. 2009;33:717–726. 19217333 10.1016/j.molcel.2009.01.026 PMC2696186

[33] Saha S , Murthy S , Rangarajan PN . Identification and characterization of a virus‐inducible non‐coding RNA in mouse brain. J Gen Virol. 2006;87:1991–1995. 16760401 10.1099/vir.0.81768-0

[34] Ji P , Diederichs S , Wang W , et al. MALAT‐1, a novel noncoding RNA, and thymosin beta4 predict metastasis and survival in early‐stage non‐small cell lung cancer. Oncogene. 2003;22:8031–8041. 12970751 10.1038/sj.onc.1206928

[35] Wilusz JE , Freier SM , Spector DL . 3′ end processing of a long nuclear‐retained noncoding RNA yields a tRNA‐like cytoplasmic RNA. Cell. 2008;135:919–932. 19041754 10.1016/j.cell.2008.10.012 PMC2722846

[36] Arun G , Aggarwal D , Spector DL . MALAT1 long non‐coding RNA: Functional implications. Non‐coding RNA. 2020;6:22. 32503170 10.3390/ncrna6020022 PMC7344863

[37] Cory S , Graham M , Webb E , Corcoran L , Adams JM . Variant (6;15) translocations in murine plasmacytomas involve a chromosome 15 locus at least 72 kb from the c‐myc oncogene. EMBO J. 1985;4:675–681. 3924592 10.1002/j.1460-2075.1985.tb03682.x PMC554241

[38] Graham M , Adams JM . Chromosome 8 breakpoint far 3′ of the c‐myc oncogene in a Burkitt's lymphoma 2;8 variant translocation is equivalent to the murine pvt‐1 locus. EMBO J. 1986;5:2845–2851. 3024964 10.1002/j.1460-2075.1986.tb04578.x PMC1167233

[39] Cho SW , Xu J , Sun R , et al. Promoter of lncRNA gene PVT1 is a tumor‐suppressor DNA boundary element. Cell. 2018;173:1398–1412.e22. 29731168 10.1016/j.cell.2018.03.068 PMC5984165

[40] Onagoruwa OT , Pal G , Ochu C , Ogunwobi OO . Oncogenic role of PVT1 and therapeutic implications. Front Oncol. 2020;10:17. 32117705 10.3389/fonc.2020.00017 PMC7010636

[41] Hezroni H , Ben‐Tov Perry R , Gil N , Degani N , Ulitsky I . Regulation of neuronal commitment in mouse embryonic stem cells by the Reno1/Bahcc1 locus. EMBO Rep. 2020;21:e51264. 32969152 10.15252/embr.202051264 PMC7645239

[42] Rontani P , Perche O , Greetham L , et al. Impaired expression of the COSMOC/MOCOS gene unit in ASD patient stem cells. Mol Psychiatry. 2021;26:1606–1618. 32327736 10.1038/s41380-020-0728-2 PMC8159765

[43] Lyu Y , Jia W , Wu Y , et al. Cpmer: A new conserved eEF1A2‐binding partner that regulates *Eomes* translation and cardiomyocyte differentiation. Stem Cell Rep. 2022;17:1154–1169. 10.1016/j.stemcr.2022.03.006 PMC9133893 35395174

[44] Ghildiyal R , Sawant M , Renganathan A , et al. Loss of Long noncoding RNA NXTAR in prostate cancer augments androgen receptor expression and enzalutamide resistance. Cancer Res. 2022;82:155–168. 34740892 10.1158/0008-5472.CAN-20-3845 PMC8732311

[45] DiStefano MT , Goehringer S , Babb L , et al. The gene curation coalition: A global effort to harmonize gene‐disease evidence resources. Genet Med. 2022; S1098‐3600(22)00746‐8. 10.1016/j.gim.2022.04.017 PMC7613247 35507016

[46] Merola R , Tomao L , Antenucci A , et al. PCA3 in prostate cancer and tumor aggressiveness detection on 407 high‐risk patients: A National Cancer Institute experience. J Exp Clin Cancer Res. 2015;34:15. 25651917 10.1186/s13046-015-0127-8 PMC4324853

[47] Zhao T , Zhang T , Zhang Y , Zhou B , Lu X . Paclitaxel resistance modulated by the interaction between TRPS1 and AF178030.2 in triple‐negative breast cancer. Evid Based Complement Alternat Med. 2022;2022:6019975. 35399640 10.1155/2022/6019975 PMC8986375

[48] Xu Y , Yu X , Xu J , et al. LncRNA RP11‐138J23.1 contributes to gastric cancer progression by interacting with RNA‐binding protein HuR. Front Oncol. 2022;12:848406. 35392234 10.3389/fonc.2022.848406 PMC8980803

[49] Wang Z , Cao L , Zhou S , Lyu J , Gao Y , Yang R . Construction and validation of a novel Pyroptosis‐related four‐lncRNA prognostic signature related to gastric cancer and immune infiltration. Front Immunol. 2022;13:854785. 35392086 10.3389/fimmu.2022.854785 PMC8980360

[50] Zhou H , Huang X , Shi W , Xu S , Chen J , et al. LncRNA RP3‐326I13.1 promotes cisplatin resistance in lung adenocarcinoma by binding to HSP90B and upregulating MMP13. Cell Cycle. 2022;21:1–15. 35298351 10.1080/15384101.2022.2051971 PMC9345617

[51] Stein CS , Jadiya P , Zhang X , et al. Mitoregulin: A lncRNA‐encoded microprotein that supports mitochondrial Supercomplexes and respiratory efficiency. Cell Rep. 2018;23:3710–3720.e8. 29949756 10.1016/j.celrep.2018.06.002 PMC6091870

[52] Prensner JR , Enache OM , Luria V , et al. Noncanonical open reading frames encode functional proteins essential for cancer cell survival. Nat Biotechnol. 2021;39:697–704. 33510483 10.1038/s41587-020-00806-2 PMC8195866

[53] D'Lima NG , Ma J , Winkler L , Chu Q , Loh KH , et al. A human microprotein that interacts with the mRNA decapping complex. Nat Chem Biol. 2017;13:174–180. 27918561 10.1038/nchembio.2249 PMC5247292

[54] Eckhart L , Lachner J , Tschachler E , Rice RH . TINCR is not a non‐coding RNA but encodes a protein component of cornified epidermal keratinocytes. Exp Dermatol. 2020;29:376–379. 32012357 10.1111/exd.14083 PMC7187231

[55] Nita A , Matsumoto A , Tang R , et al. A ubiquitin‐like protein encoded by the “noncoding” RNA TINCR promotes keratinocyte proliferation and wound healing. PLoS Genet. 2021;17:e1009686. 34351912 10.1371/journal.pgen.1009686 PMC8341662

[56] Chen Y , Li X , Li B , et al. Long non‐coding RNA ECRAR triggers post‐natal myocardial regeneration by activating ERK1/2 signaling. Mol Ther. 2019;27:29–45. 30528086 10.1016/j.ymthe.2018.10.021 PMC6319349

